# A three-phase *in-vitro *system for studying *Pseudomonas aeruginosa *adhesion and biofilm formation upon hydrogel contact lenses

**DOI:** 10.1186/1471-2180-10-282

**Published:** 2010-11-09

**Authors:** Claudia Rändler, Rutger Matthes, Andrew J McBain, Bernd Giese, Martin Fraunholz, Rabea Sietmann, Thomas Kohlmann, Nils-Olaf Hübner, Axel Kramer

**Affiliations:** 1 Department of Hygiene and Environmental Medicine, Ernst Moritz Arndt University Greifswald, Greifswald, Germany; 2 School of Pharmacy and Pharmaceutical Sciences, The University of Manchester, Manchester, UK; 3 Competence Center for Functional Genomics, Ernst Moritz Arndt University Greifswald, Greifswald, Germany; 4 Department of Microbiology, Ernst Moritz Arndt University Greifswald, Greifswald, Germany; 5 Institute of Community Medicine, Ernst Moritz Arndt University Greifswald, Greifswald, Germany

## Abstract

**Background:**

*Pseudomonas aeruginosa *is commonly associated with contact lens (CL) -related eye infections, for which bacterial adhesion and biofilm formation upon hydrogel CLs is a specific risk factor. Whilst *P. aeruginosa *has been widely used as a model organism for initial biofilm formation on CLs, *in-vitro *models that closely reproduce *in-vivo *conditions have rarely been presented.

**Results:**

In the current investigation, a novel *in-vitro *biofilm model for studying the adherence of *P. aeruginosa *to hydrogel CLs was established. Nutritional and interfacial conditions similar to those in the eye of a CL wearer were created through the involvement of a solid:liquid and a solid:air interface, shear forces and a complex artificial tear fluid. Bioburdens varied depending on the CL material and biofilm maturation occurred after 72 h incubation. Whilst a range of biofilm morphologies were visualised including dispersed and adherent bacterial cells, aggregates and colonies embedded in extracellular polymer substances (EPS), EPS fibres, mushroom-like formations, and crystalline structures, a compact and heterogeneous biofilm morphology predominated on all CL materials.

**Conclusions:**

In order to better understand the process of biofilm formation on CLs and to test the efficacy of CL care solutions, representative *in-vitro *biofilm models are required. Here, we present a three-phase biofilm model that simulates the environment in the eye of a CL wearer and thus generates biofilms which resemble those commonly observed *in-situ*.

## Background

The use of contact lenses (CLs) is a major risk factor for the development of microbial keratitis [1-3]. Whilst Gram-negative bacteria, particularly *P. aeruginosa*, are commonly associated with the condition, within the last four years, two notable outbreaks of CL-associated infectious keratitis have occurred, which were caused by the normally uncommon agents, *Fusarium *(2006 in Singapore, Hong Kong and the USA) and *Acanthamoeba *(2007 in USA). These infections were associated with the use of the CL care solutions "ReNu_® _with MoistureLoc^®^" and "Complete^® ^MoisturePlus™", respectively [4].

The ability of microorganisms to adhere to CL surfaces and to form biofilms plays an important role in the development of CL-related eye infections [5]. Bacterial adhesion and the associated infection risk are influenced by a combination of different factors which include: i. the composition of an individual's tear fluid (organic and inorganic substances) [6]; ii. environment (weather, temperature, air pollution) [7]; iii. CL composition (material, water content, ionic strength) [8]; iv. the nature and quantity of the microbial challenge (species, strain) [8]; v. wearer habits (such as swimming and sleeping during CL wear) [9]; and vi. CL hygiene (CL care solution and CL handling) [7,10-12]. Furthermore, biofilms are a risk factor for concomitant infections with other microorganisms, including *Acanthamoeba*, which can co-exist synergistically with *P. aeruginosa *in biofilms, resulting in an increased risk of *Acanthamoeba *keratitis [13]. Biofilm formation on CLs is therefore a complex process which may differ markedly between individuals.

One of the most common organisms associated with bacterial adhesion to CLs and with CL-related eye infections is *P. aeruginosa *[10,14]. *P. aeruginosa *is commonly isolated from soil and aquatic environments, is well adapted to survive in water and aqueous eye-products [14], and, through a number of physiological adaptations is generally recalcitrant and can often survive exposure to enzymatic CL care products [15]. As a versatile opportunistic pathogen, it is frequently associated with corneal ulcers. *P. aeruginosa *is accordingly a commonly studied model organism for the *in-vitro *investigation of biofilm formation on CLs [8,13,16-31]. Most previous *in-vitro *studies of biofilm formation on CLs have focused on initial bacterial adherence; only a limited number of reports have described models designed to maximise validity in investigations of the anti-biofilm efficacy of CL solutions [32,33]. With respect to simulating the milieu of the human eye, studies which have utilised saline omit important factors which may promote biofilm development [13,23-29]. Hence, there is a need for *in-vitro *biofilm models that more closely mimic the conditions in the eye of a CL wearer. Such models may contribute to understanding the complex process of *in-vivo *biofilm formation and facilitate the evaluation of the anti-biofilm efficacy of CL care solutions. Data thus generated can be used to calculate and minimise the risk of microbe-associated and CL-related eye diseases. The aim of the current study therefore, was to develop a realistic *in-vitro *biofilm model for the bacterial adhesion of *P. aeruginosa *to hydrogel CLs under conditions which resemble the environment in the eye of a CL wearer. Bacterial adherence was evaluated over time by counting colony forming units (CFUs). The morphology and composition of the biofilms were analysed by confocal laser scanning and scanning electron  microscopy.

## Methods

### Contact lenses

Four different hydrogel CLs were studied corresponding to the FDA Groups (FDA Group 1: non-ionic, low water (<50% H_2_O); FDA Group 2: non-ionic, high water (>50% H_2_O); and FDA Group 4: ionic, high water (>50% H_2_O)). The CLs examined in this study are described in detail in Table 1. CLs of the minor FDA Group 3 (ionic/low water) were not included in this study, because the physicochemical properties of these CLs are similar to that of the FDA Group 4. Instead, two widely used silicone hydrogel CLs (FDA Group 1) with different characteristics were selected. In all cases, unused CLs were removed from the original package and washed with sterile isotonic saline prior to use in the biofilm model. For the sake of consistency, all CLs exhibited a power of -3.00 dioptre.

**Table 1:** Properties of hydrogel contact lenses used in this study

Proprietary name	ACUVUE 2	PROCLEAR	BIOFINITY	AIROPTIX
United States Adopted Name (USAN)	Etafilcon A	Omafilcon A	Comfilcon A	Lotrafilcon B
Manufacturer	Johnson & Johnson	Cooper Vision	Cooper Vision	CIBA Vision
Water content (%)	58	62	48	33
Ionic charge	Ionic	Non-ionic	Non-ionic	Non-ionic
Oxygen permeability (Dk)	22	27	128	110
Centre thickness (mm) -3.00 D	0.084	0.065	0.08	0.08
Oxygen transmissibility (Dk/t) at 35°C	33.3	42	160	138
Basis curve (mm)	8.7	8.6	8.6	8.6
Diameter (mm)	14.0	14.2	14.0	14.2
Surface treatment	None	None	None	25-nm-thick plasma coating with high refractive index
FDA Group	4 (Conventional hydrogel)	2 (Conventional hydrogel)	1 (Silicone hydrogel)^α^	1 (Silicone hydrogel)^β^
Replacement and wearing schedule*	Every 2 weeks (daily wear) *OR *six nights extended wear	Every 4 weeks (daily wear)	Every 4 weeks (daily, continuous *OR *flexible wear)	Every 4 weeks (daily wear) *OR *up to six nights extended wear
Principal monomers	HEMA, MA	HEMA, PC	FM0411M, HOB, IBM, M3U, NVP, TAIC, VMA	DMA, TRIS, siloxane monomer

HEMA (poly-2-hydroxyethyl methacrylate); MA (methacrylic acid); PC (phoshoryl choline); DMA (N,N-dimethylacryl amide); TRIS (trimethylsiloxy silane); DMA, N,N-dimethylacrylamide; FM0411M (α-methacryloyloxyethyl iminocarboxyethyloxypropyl-poly(dimethylsiloxy)-butyldimethylsilane); HOB (2-hydroxybutyl methacrylate); IBM (isobornyl methacrylate); M3U αω -bis(methacryloyloxyethyl iminocarboxy ethyloxypropyl)-poly(dimethylsiloxane)-poly(trifluoropropylmethylsiloxane)-poly(ω methoxy- poly(ethyleneglycol)propylmethylsiloxane); NVP (N-vinyl pyrrolidone); TAIC (1,3,5-triallyl-1,3,5-triazine-2,4,6(1H,3H,5H)-trione); VMA (N-Vinyl-N-methylacetamide)

^α ^third silicone generation

^β ^first silicone generation

*It is recommended that the CL wearer first be evaluated on a daily wear schedule. If successful, then a gradual introduction of extended wear can be followed as determined by the prescribing Eye Care Practitioner.

### Artificial tear fluid

A mixture of human blood serum (20% v/v) and lysozyme (2 g/L, Sigma Aldrich, Steinheim, Germany) diluted in an ocular irrigation solution BSS^® ^(balanced salt solution, Delta Select GmbH, Dreieck, Germany) was used as artificial tear fluid. Human blood serum was obtained from healthy blood donors in the Department of Transfusion Medicine of the University of Greifswald (Germany). Donors gave informed consent to provide an additional blood sample of 8 mL whole blood for research purposes. The serum samples were collected in 50 mL tubes and stored at -20°C.

### Test bacterium and growth conditions

The mucoid environmental *P. aeruginosa *strain SG81, previously isolated from a biofilm in a technical water system, was kindly supplied by Prof. Dr. Hans-Curt Flemming (Biofilm Center, Duisburg, Germany) and stored at -20°C. The test bacterium was grown on Columbia blood agar (BD, Heidelberg, Germany) for 24 h at 37°C. Thereafter, a single colony was inoculated onto a trypticase soy agar plate (TSA, Oxoid, Wesel, Germany) and was incubated for 24 h at 37°C. In order to prepare a washed cell inoculum for the biofilm model, the colonies were harvested from the agar plate by scraping with a Spatula Drigalski and suspended in 10 mL PBS (pH 7.2; 0.1418 mol/L NaCl, 0.0030 mol/L KCl, 0.0067 mol/L Na_2_HPO_4 _and 0.0016 mol/L KH_2_PO_4_). Harvested bacteria were then washed twice by centrifugation for 15 min at 3000 × g, the resuspension in 5 mL ocular irrigation solution BSS^® ^to yield a final concentration of 1 × 10^10 ^CFU/mL which was verified by colony-counting as outlined below.

### Bacterial adhesion studies with the three-phase biofilm model

**Figure 1:**
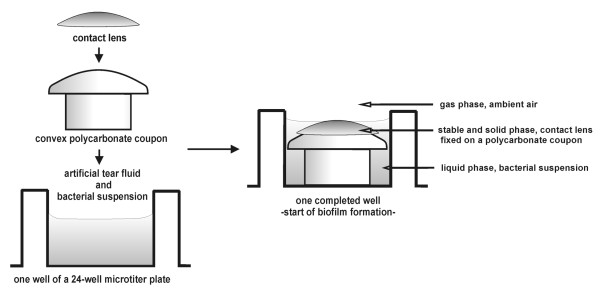
Assembly of the *in-vitro *three-phase biofilm model.

The biofilm model was housed and replicated within in a 24-well microtiter plate (Sarstedt, Nümbrecht, Germany). Convex polycarbonate coupons (PCs, in-house production) were used as the contact surface for the CLs and were placed in the wells (Figure 1). The bacterial suspension, consisting of the artificial tear fluid and the bacterial cells in a ratio of 5:1 was adjusted to a final concentration of approximately 1.0 × 10^9 ^CFU/mL. CLs were placed convex side up on the top of the PCs in the wells of the microtiter plate, each well containing 1 mL of the bacterial suspension as illustrated in Figure 1. The CLs were incubated with an agitation of 240 rpm at room temperature.

### Determination of the biofilm growth on contact lenses

The CLs were incubated in the biofilm model for 2, 4, 8, 12, 24, 36, 48 and 72 h. After incubation, CLs were carefully removed at the indicated times and gently washed in PBS. To harvest the biofilm from the CL surface, vortex agitation in the presence of glass beads (2 mm Ø) was performed for 2 min. This regimen has been found to effectively remove adhered bacteria without significantly reducing their viability. After removal, viable cells were quantified using colony counting in log serial dilutions of the homogenate. Two aliquots of each dilution were plated on trypticase soy agar plates and incubated for 24 h at 37°C. This adherence assay was performed in quadruplicate for each incubation time and for each CL material. The results were reported in log transformations of the CFUs per surface area of the CL (log [CFU/cm^2^]).

### Confocal laser scanning microscopy

Biofilm samples were visualised using a ZEISS LSM 510 META confocal laser scanning microscope (CLSM510, Zeiss, Jena, Germany). Microscopic observations were performed using a Plan-Neofluar 40× oil immersion objective with a numerical aperture of 1.3. Confocal images, unless noted otherwise, represent 1-μm-thick confocal slices of the specimen. Non-confocal, transmitted light images were generated by the longest excitation wavelength of the respective multi-track channel combination and a transmitted-light detector below the specimen/focal plane.

Following incubation, the washed CL samples were transferred to a 24-well microtiter plate and incubated immediately with one of four dyes (Table 2). CTC was used for determining the respiratory activity and viability of the bacterial cells. The reduction of CTC by the respiratory electron transport chain of viable bacterial cells leads to insoluble, fluorescent formazan crystals (CTF) [34]. Concanavalin (Con) A (a lectin) conjugated with the fluorescent substance Alexa Fluor 488 was used to visualise polysaccharides: when Con A Alexa Fluor 488 is intercalated into the glucose and mannose residues of polysaccharides, green fluorescence signals are emitted [35]. Even though Con A intercalates mainly into reducing sugars, Wingender et al. [35,36] have observed that it is also suitable for the visualisation of alginate within the EPS of the strain *P. aeruginosa *SG81. Acridine orange is a nucleic-acid selective fluorescent dye and interacts with DNA and RNA by intercalation and electrostatic attractions, respectively [37]. DAPI exhibits a particular affinity to double-stranded DNA and is considerably more intensively fluorescent in the intercalation state [38]. An advantage of DAPI is that it can be used concurrently with CTC, due to their different emission ranges, whereas acridine orange exhibits nearly the same emission range as CTC (Table 2).

**Table 2:** Characteristics of the fluorescent dyes used in confocal laser scanning microscopy

Fluorescent substance	Manufacturer	Excitation wavelength (Laser) in [nm]	Emission range in [nm]	Concentration/incubation time/temperature	Fluorescence of
**Acridine orange**	Acridine orange - zinc chloride, Applichem GmbH, Darmstadt; Germany	Argon 458	505-550 BP 592-753 BP	200 μg/mL; 2-5 min; RT	nucleic acids
**DAPI**	Dapi Biochemica, Applichem GmbH, Darmstadt; Germany	Diode 405	420-480 BP	20 μg/mL; 30 min; RT	nucleic acids
**ConA-Alexa Fluor 488**	Concanavalin A - Alexa Fluor^® ^488 conjugated, Invitrogen Molecular Probes, Eugene, USA	Argon 488	505-530 BP	10 μg/mL; 30 min; RT	polysaccharides
**CTC**	CTC (5-Cyano-2,3-di-4-tolyl-tetraolium chloride), Polysciences Inc.; Warrington, USA	Diode 561	575 LP	1.25 mg/mL; 3 h; RT	redox activity

After incubation, an effective washing and preparation method was necessary, because dyes stain not only into the biofilm matrix but also into the CL material, which may produce strong background fluorescence. Therefore, the samples were rinsed at least five times with PBS to reduce background fluorescence. After staining and washing, the CL samples were placed onto glass slides, embedded in 10 μL Mowiol 4-88 (Polysciences Inc., Warrington, USA) and covered with a cover slip for observation by CLSM.

### Scanning electron microscopy (SEM)

*P. aeruginosa *adhesion to CLs was also observed by SEM (DSM-940A, Zeiss, Oberkochen, Germany) at various magnifications (100×, 500×, 2000×, 5000×). All buffer solutions were passed through 0.2 μm filters to eliminate background particles. The CL samples were fixed in HEPES buffer (10 mM, pH 7.4) containing NaN_3 _(50 mM), 3% glutaraldehyde, and 4% paraformaldehyde for 1 h at room temperature and then overnight at 4°C.

Further treatment was carried out using two different methods. They were: i. critical point drying, which consisted of 2% tannic acid for 1 h, 1% osmium tetroxide for 2 h, 1% thiocarbohydrazide for 30 min, 1% osmium tetroxide overnight, and 2% uranyl acetate for 2 h, with washing steps in between. The samples were then dehydrated by immersion in increasing concentrations of ethanol (10 - 100%) and dried in a critical point drier using amylacetate and liquid CO_2_; ii. sodium hydroxide drying: osmium tetroxide vapor for 3 days; drying over sodium hydroxide disks for 3 weeks at -20°C. All samples were mounted onto aluminum stubs and sputter-coated with gold for observation using SEM.

### Statistical analyses

Statistical analyses were performed using analysis of variance (ANOVA) to determine the main effects of CL material and incubation time, and the interaction effect on biofilm growth in (log [CFU/cm^2^]). Additionally, ANOVA was performed with Tukey's HSD post-hoc test to compare the viable bacterial cell counts in log [CFU/cm^2^]. Two distinct comparisons were made: i. differences between the viable cell counts at different incubation times (24, 48 and 72 h) independent of the CL materials and separately for each CL material; ii. differences between the viable cell counts on various CL materials independent of the incubation times and separately for each incubation time. P ≤ 0.05 was considered statistically significant.

## Results

### *Pseudomonas aeruginosa* biofilm growth on various contact lens materials

**Figure 2:**
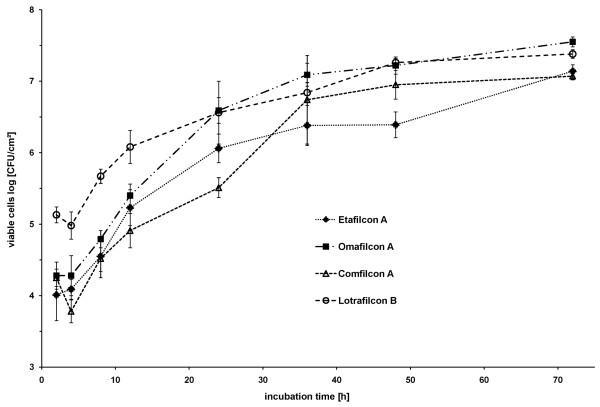
**Biofilm growth dynamics on contact lens materials. **Curves represent the means of data in log [CFU/cm^2^]; all test were performed in quadruplicate (± standard deviation).

**Figure 3:**
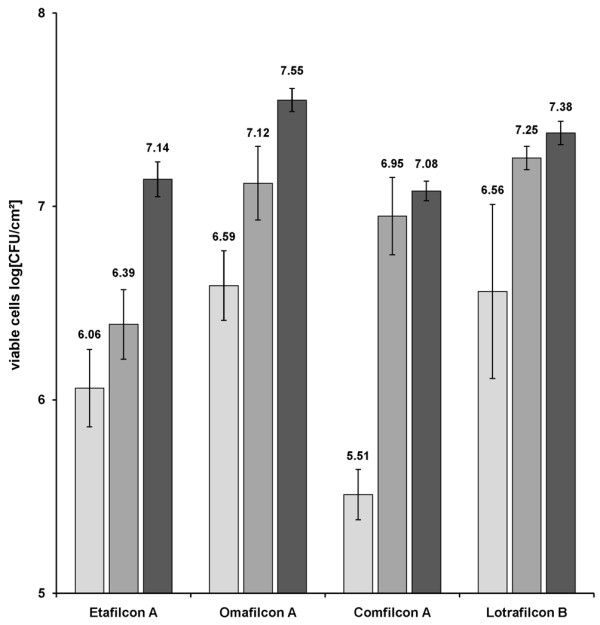
***P. aeruginosa *biofilm cell counts for various contact lens materials after 24, 48 and 72 h of growth. **Results are the means of data performed in quadruplicate (± standard deviation) in log [CFU/cm^2^] at the different incubation times: 24 h (light grey), 48 h (middle grey) and 72 h (dark grey).

To evaluate biofilm formation in the novel *in-vitro *biofilm model (Figure 1), the accumulation of viable bacterial cells over time was measured on four CLs using quantitative culturing (Figure 2). For comparison and for statistical analysis, variation between the CL materials in terms of viable cell counts in log [CFU/cm^2^] after 24, 48 and 72 h growth are represented separately in Figure 3. Analysis of variance showed that biofilm growth was significantly affected primarily by the incubation time, and secondarily by the CL material. The interaction effect of time and material had a comparatively minor effect (Table 3).

**Table 3:** Results of analysis of variance: main effects of contact lens material and incubation time and the interaction effect on bacterial adherence of *P. aeruginosa *SG81 over time

Source	Sum of Squares	DF	Mean Square	F Value	Sig.
**Contact lens material**	3.276	3	1.092	28.266	< 0.001
**Incubation time**	9.293	2	4.646	120.278	< 0.001
Contact lens material * Incubation time	1.569	6	0.261	6.769	< 0.001
**Error**	1.198	31	0.039		
**Corrected total**	15.292	42			

Although viable cell numbers significantly increased over time, independent of the CL material (Table 4), distinct patterns of growth for each CL material were observed. Biofilm formation on Etafilcon A (FDA Group 4) showed a latent phase between 2 h and 4 h, followed by continuous, rapid accumulation within 24 h, a latent phase on the second day, followed by a significant growth phase on the third day. Biofilm formation on Omafilcon A (FDA Group 2) progressed through an early latent phase in the first 4 h, followed by rapid growth to a comparatively high level of adhered cells within 24 h, and last by an intermediate phase between 24 h and 72 h with significantly decelerated growth. In contrast, biofilm formation on Comfilcon A (FDA Group 1) was characterised by a decrease in growth between 2 h and 4 h, followed by the lowest increase in growth on the first day and significant rapid growth on the second day. After 2 days, a stationary phase for biofilm formation was reached on Comfilcon A. Lotrafilcon B (FDA Group 1) also showed a decrease in growth between 2 h and 4 h, but yielded the highest initial number of adhered viable cells within 24 h growth, followed by a significant continuous increase in biofilm growth up to 48 h; a stationary phase after 2 days was also attained.

**Table 4:** Significance of the differences between the viable cell counts of *P. aeruginosa *SG81 at different incubation times

Contact lens material	Comparison of the incubation times		
	24 h - 48 h	24 h - 72 h	48 h - 72 h
**Independent**	< 0.001	< 0.001	< 0.001
**Etafilcon A**	0.084	< 0.001	0.003
**Omafilcon A**	0.004	< 0.001	0.020
**Comfilcon A**	< 0.001	< 0.001	0.435
**Lotrafilcon B**	0.041	0.020	0.868

Tukey's HSD Post-hoc test. P ≤ 0.05 was considered statistically significant.

A comparison of the viable cell counts associated with the test CL materials after 24 h showed no significant difference between the different CL materials (Table 5), due to the broad variance of the data. After 72 h however, variance was minimal and as a result, significant differences were observed between the viable cell counts of the various CLs. Accordingly, significantly more viable *P. aeruginosa *SG81 cells grew in 72 h (Figure 3) on Omafilcon A (7.55 ± 0.07 log [CFU/cm^2^]) and Lotrafilcon B (7.38 ± 0.06 log [CFU/cm^2^]) than on Etafilcon A (7.14 ± 0.09 log [CFU/cm^2^]) and Comfilcon A (7.07 ± 0.05 log [CFU/cm^2^]). Although there were differences in kinetics, biofilms grown for 72 h were used in qualitative experiments because variance in biofilm formation was minimised at this point of time, and biofilms had reached a stationary phase on most of the CL materials.

**Table 5:** Significance of the differences between the viable cell counts of *P. aeruginosa *SG81 on different CL materials

Incubation time	Contact lens material		
	2	3	4
**Independent**			
**1**	< 0.001	0.987	< 0.001
**2**	-	< 0.001	0.980
**3**	-	-	< 0.001

**24 h**			
**1**	0.070	0.057	0.093
**2**	-	0.001	0.998
**3**	-	-	0.001

**48 h**			
**1**	0.001	0.008	0.001
**2**	-	0.515	0.743
**3**	-	-	0.154

**72 h**			
**1**	< 0.001	0.601	0.006
**2**	-	< 0.001	0.033
**3**	-	-	0.001

Tukey's HSD Post-hoc test: 1. Acuvue 2 (Etafilcon A); 2. Proclear (Omafilcon A); 3. Biofinity (Comfilcon A); 4. Air Optix (Lotrafilcon B). P ≤ 0.05 was considered statistically significant.

### Characterisation of biofilms on contact lenses using CLSM and SEM

**Figure 4:**
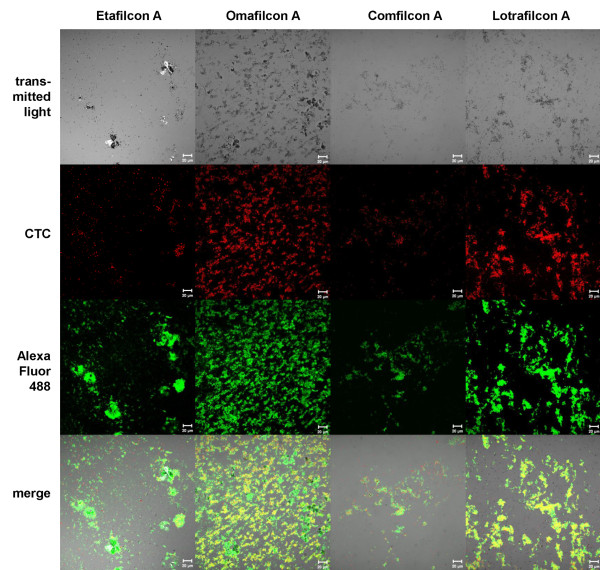
**Predominant *P. aeruginosa *biofilm structures depend on contact lens materials after 72 h growth. **Transmitted light micrographs: deposits and adherent bacterial cells on the contact lenses are visible as grey dots and shadows. CTC staining of the biofilms (red) shows the metabolic activity of viable bacteria cells. ConA Alexa Fluor 488 staining of the biofilms (green) verifies the presence of alginate within the biofilm matrix. Superimposition of the transmitted light micrographs and the fluorescence micrographs (merge) shows the correlation of the CTC and ConA Alexa Fluor 488 staining regions. Bar = 20 μm.

To characterise the predominant biofilm structures on various CL materials (Figure 4), biofilms were stained with CTC for observation of the viable bacterial cells. The biofilms of the various CL materials often showed a heterogeneous EPS structure, visible as ConA Alexa Fluor 488, green stained fluorescent, cloud-like regions. Bacterial adhesion densities on Etafilcon A and Comfilcon A were obviously lower than on Omafilcon A and Lotrafilcon B, which correlated with the findings of the viable cell count analysis.

**Figure 5:**
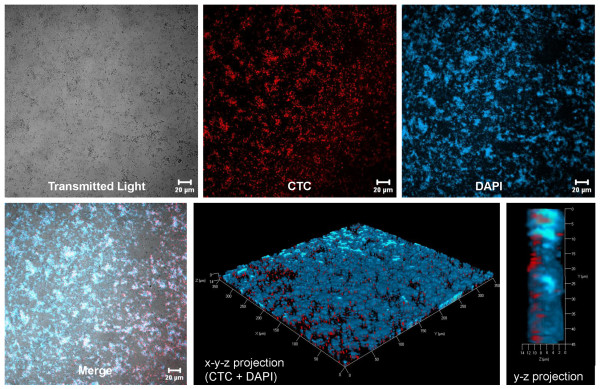
**Cells of *P. aeruginosa *SG81 adhere in patches to Lotrafilcon B after 72 h incubation. **Transmitted light micrograph: deposits and adherent bacterial cells on the contact lens are visible as grey dots and shadows. DAPI staining of the biofilm (blue) shows all adherent bacterial cells (viable and dead). CTC staining of the biofilm (red) shows the metabolic activity of the viable bacterial cells. Superimposition of the transmitted light micrograph and the fluorescence micrographs (merge) shows the correlation of the CTC and DAPI stained regions. The three-dimensional representation gives an illustration of the spatial structure and the thickness of the biofilm matrix (~12 μm). Bar = 20 μm.

**Figure 6:**
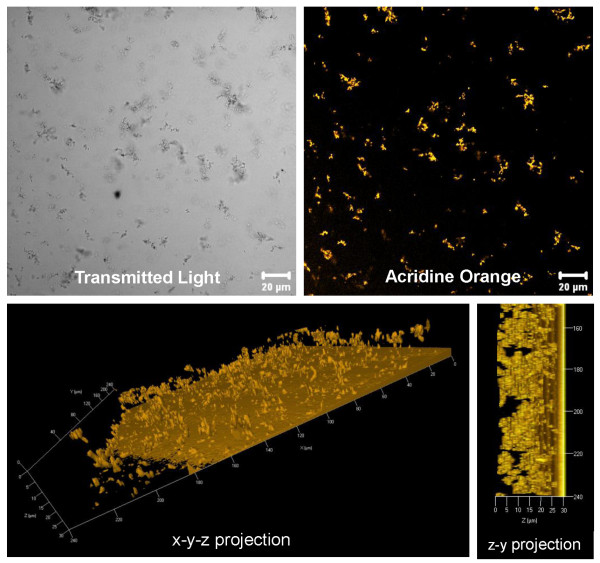
**Small colonies of *P. aeruginosa *cells are dispersed homogeneously and thinly throughout the biofilm matrix on Etafilcon A after 72 h growth. **The non-confocal transmitted light micrograph and the acridine orange stained micrograph are x-y projections of a slice of the z-stack (z = 12 μm) of the biofilm matrix. Bacterial cells were stained with the dye acridine orange to observe the total amount of bacterial cells (viable and dead). The three-dimensional representation of the biofilm stained with acridine orange illustrates the distribution of the bacterial cells throughout the biofilm matrix and the thickness of the biofilm matrix (~ 30 μm). Furthermore, the fluorescent dye acridine orange intercalates not only into nucleic acids but also into the contact lens hydrogel polymer matrix.

**Figure 7:**
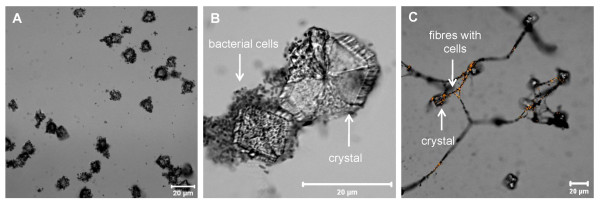
**Various, rarely observed biofilm structures such as crystals, granular materials and fibres on the air-exposed contact lens surface after 72 h growth. **Extensive agglomerations of bacterial cells were found to adhere to the surface of crystals and granular materials. Crystals and granular materials were also associated with the formation of fibres. Acridine orange staining of the fibres verifies the presence of bacterial cells throughout the fibres. Bar = 20 μm.

Among the observed, predominant biofilm morphologies, various structures were characterised, independent of the CL material. For example, Figure 5 depicts a heterogeneous biofilm stained with DAPI and CTC for examining the proportion of total and viable bacterial cells. A comparison of DAPI and CTC fluorescent regions showed that most of the cells were viable. Additionally, *P. aeruginosa *SG81 biofilms were found to occur either in a homogeneous, thin, dispersed structure (Figure 6) or in a more heterogeneous, compact form (Figure 5). Whilst both structures were found on every CL, the heterogeneous form was predominant. Furthermore, the architecture of the biofilms formed at the periphery of the CLs differed from those at the centre. Specifically, the central air-exposed region was characterised by crystalline and granular structures (Figure 7) which were often surrounded by agglomerations of bacterial cells. Other biofilm structures, such as the formation of fibres between crystals, were only rarely found. Bacterial cells embedded along the fibres were apparent following acridine orange staining.

**Figure 8:**
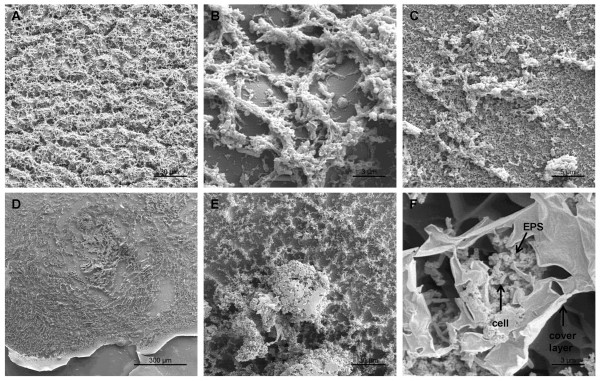
**Observation of various biofilm structures using SEM techniques after 72 h incubation. **Biofilms in A-C were prepared using the SEM method with critical point drying. Biofilms in D-F were prepared using the SEM method with prolonged sodium hydroxide drying. Etafilcon A: A (500×), B (5000×), D (100×); Omafilcon A: C (2000×), E (500×), F (5000×). Different structural formations appear to cover the contact lens surface: extensive networks consisting of EPS and bacterial cells, mushroom-like structure, clumps and cover layers overlap compact, thick agglomerations of cells which are embedded in a network of EPS.

Various biofilm structures were also observed by SEM (Figure 8). SEM micrographs of samples prepared according to the method of dehydration by immersion in increasing concentrations of ethanol followed by critical point drying depicted networks of EPS formations with fibres and clumps. Ethanol preparation led to denaturation of proteins within the EPS, resulting in a clear visualisation of exposed bacterial cells (Figure 8A-C). When SEM samples were prepared according to the method of prolonged sodium hydroxide drying without denaturation, a thick, mature biofilm consisting of clumps, mushroom-like formations and networks of EPS and bacterial cells was observed (Figures 8D-F). Other structures such as a conditioning film covering the CL surface or a cover layer overlapping the biofilm matrix were also observed (Figures 8D and 8F).

## Discussion

Several biofilm models have previously been used to investigate bacterial adhesion upon CLs, mainly in planktonic suspensions in microtiter plates [13,19,28-32] or by suspending CLs in culture vessels [8,16,17,24,26,27,39-41]. Another approach, which provides a continuous nutrient supply, involves the location of CL materials into flow cells [20-23,42]. These biofilm models are predominantly two-phase systems, since they provide a solid:liquid interface and furthermore, in the absence of a support system, the convex surface curvature of the CL is likely to vary significantly with loss of the normally convex surface tension, for example within flow cells and other model systems due to fluid dynamic forces. Although these *in-vitro *biofilm models are useful for obtaining information about the characteristics of bacterial adhesion on CL surfaces, it is suggested that the elaborations presented in the current study provide a greater degree of realism. These are i. the use of a mucoid, environmental bacterial strain, ii. the use of a complex artificial tear fluid, iii. the incorporation of a convex contact surface to stabilise the convex shape of the CL, in a manner analogous to that of the human cornea, iv. exposure of the solid substratum (i.e. the CL) to both, liquid and air, phases and v. the simulation of eyelid movements.

Given that suboptimal use and care of CLs is known to be common [43-45] among CL wearers, the model described in the current study was designed to produce mature, recalcitrant biofilms which reproduce the morphology and importantly, the resistance properties of real-life ocular biofilms that can occur following incorrect wearing schedules, and ineffective CL care. *P. aeruginosa *SG81 is a stable, alginate-producing strain that forms strongly mucoid colonies on standard media agar [35,46] and has been previously validated as model organism for investigation of *in-vitro *biofilm formations [35,36,47,48]. With this strain, morphologically mature biofilms were generated on every test CL material.

With respect to growth media, the majority of previous studies have reported the use of nutritionally inert PBS suspensions [13,23-28,32,39,49] or have used simple proteins such as lysozyme [17] or albumin [31] in aqueous solution. More complex artificial tear fluids have also been developed [8,16,19,30] consisting of for example, a mixture of turkey egg white lysozyme, immunoglobulin A from human colostrum, bovine lactoferrin, serum albumin and mucin [16]. Since natural tear fluid and human blood serum show marked similarities in pH value, osmolarity, ionic strength, and protein composition [6,50-52], the artificial tear fluid used in the current investigation offers a relatively high degree of realism. Because of their similarities, human blood serum has been previously used clinically as a replacement for human tear fluid [52-54]. Although human blood serum represents a useful analogue of human tear fluid, serum has a higher protein concentration, lower quantities of antimicrobial substances, and lacks tear-specific proteins. In the current investigation therefore, the protein concentration of serum was reduced to a physiologically relevant value by diluting 1:5 with the ocular irrigation solution BSS^® ^and the tear-specific protein lysozyme was added at a physiological concentration. The serum used was pooled and aliquotted from 50 different patient samples and thus avoids *in-vivo *variation between single serum samples.

To prevent the deformation of the flexible CL caused by floating loosely in a suspension that presumably is a common feature of previously reported models, supportive coupons incorporating convex contact surfaces were machined from polycarbonate. The resulting support of the CLs resulted in a stable, solid surface with a high surface tension incident to the convex shape of the CL. Additionally, intermittent contact with air for the central section of the CL was achieved by the use of continuous rotational mixing, combined with adjustment of the volume of artificial tear fluid so that the top of the CL surface was in contact with air in a manner similar to that which occurs *in-vivo *through the movement of the eyelid (Figure 1). Continuous agitation also effectively avoided dehydration of CLs. The effect of the third phase, forming a solid:air interface, and eyelid movements on bacterial adhesion to CLs has infrequently been reported in literature [21,24,30,55]. Vermeltfoort et al. [21] passed air bubbles over the CL to mimic the natural shear action of blinking of the eyelid. Borazjani et al. [24] proposed that the effect of tear flow and the shear force of blinking may limit bacterial development on worn CLs.

In the current study, viable bacterial numbers on the silicone CLs decreased within the first few hours, an observation that contrasts with some previous studies [19,25,26,33,56], which have generally reported a continuous increase of initial bacterial adherence. One possible explanation for this is that, in contrast to many previous studies, the artificial tear fluid used here contained antimicrobial components such as lysozyme, which may lead to an initial viable cell reduction before physiological adaptation of the bacteria could occur. Furthermore, in the current investigation, biofilms grew significantly in the first 48 h, and maturation and decelerated growth were not observed until then. In contrast, Stapleton et al. [26] reported maximal adherence after 45 min, followed by a decrease in growth and Andrews et al. [57] reported maximum adhesion following 4 h incubation. The results in the current study suggest that the conditions of the novel three-phase biofilm model may lead to slower growth over time, and the compounds of the artificial tear fluid may limit doubling times to rates more congruent with those expected *in-vivo*.

With respect to visualisation of CL biofilms, the formation of diverse, heterogeneous *P. aeruginosa *biofilms has been commonly reported. Stapleton et al. [26] for example, observed a thin sheet of fixed material on the surface of the CL that was associated with "headed-up" granular material adjacent to adhered bacteria. Other studies have noted large bacterial cell colonies on CL surfaces [22,24] or bacterial cells adhered in aggregates or clumps and stuck to EPS on albumin-coated CLs [31]. However, biofilms observed in the current study were generally more compact and extensive than in previous studies and were associated with large quantities of EPS.

Importantly, biofilm structures generated in the current model exhibit several similarities to those reported in an *in-vivo *study by McLaughlin-Borlace et al. [58] where biofilms developed various structures including clumps and networks of bacterial cells, embedded in EPS, together with thick, multilayered biofilms. The formation of a conditioning film or cover layer structures on the CL surfaces, as observed in this investigation has also been often reported in *in-vivo *studies [59-62]. Other biofilm structures, such as crystal formations, have also been observed *in-vivo *[63] and *in-vitro *[64,65]. Such similarities suggest that the three-phase biofilm model represents an improvement on two-phase systems.

## Conclusion

For standardised, realistic biofilm tests, an effective *in-vitro *model is required which closely mimics the *in-vivo *conditions of CL wear. The current study has demonstrated that growth of *P. aeruginosa *SG81 in the three-phase *in-vitro *biofilm model can simulate worst-case CL use conditions. Whilst a variety of biofilm morphological structures was observed, a compact and heterogeneous biofilm morphology predominated. Further investigations are needed to determine whether the biofilms can be standardised in order to utilise the model for the evaluation of the anti-biofilm efficacy of CL care solutions.

## Authors' contributions

CR, NOH, and AK designed the study. AK coordinated the study. CR and RM performed the adhesion assays. CLSM was performed by CR, BG, and RM. RS performed SEM. TK and CR was responsible for statistical analysis and interpretation of the data. CR and AJM wrote the manuscript and RM, BG, MF, RS, TK, NOH and AK were involved in drafting the manuscript and revising it critically for important intellectual content. All authors have read and approved the final manuscript.
